# Bias, Burnout, and Imposter Phenomenon: The Negative Impact of Under-Recognized Intersectionality

**DOI:** 10.1089/whr.2021.0138

**Published:** 2021-12-27

**Authors:** Kelly A. Cawcutt, Pauline Clance, Shikha Jain

**Affiliations:** ^1^ Division of Infectious Diseases, University of Nebraska Medical Center, Omaha, Nebraska, USA.; ^2^ Department of Psychology (Emerita), George State University, Atlanta, Georgia, USA.; ^3^ Division of Hematology and Oncology, University of Illinois at Chicago, Chicago, Illinois, USA.

## Introduction

High rates of burnout are common in health care; across specialties, disciplines, and levels of training; with ubiquitous reports of a “burnout epidemic.” Worse, burnout may be increasing amid the COVID-19 pandemic and related health care challenges. Recent statistics show that >50% of physicians and 30% of nurses experience symptoms of burnout.^1^ Similarly, incidences of implicit and explicit biases, and impostor phenomenon (IP), are also reported at high levels across health care. However, the literature examining these three phenomena predominantly describes them as separate entities. They are siloed and approached as independent issues that plague health care workers.

**Fig. 1.**
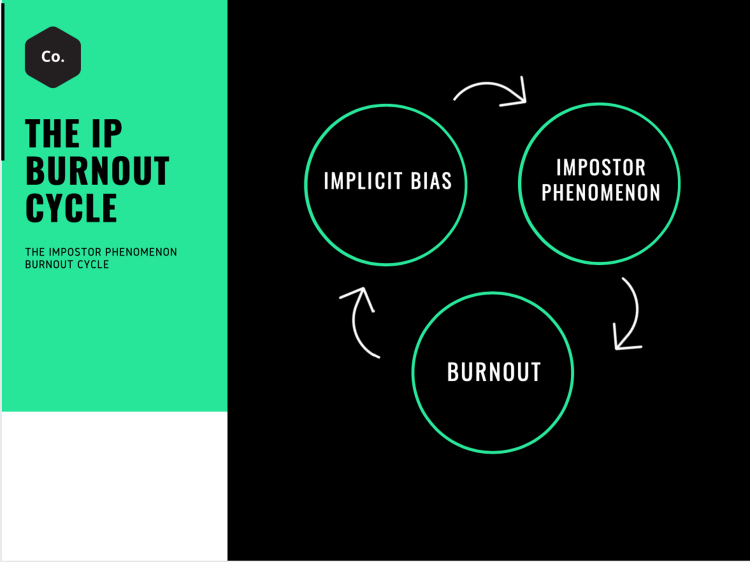
Cycle diagrams shows the interplay between implicit bias, impostor phenomenon and burnout.

In reality, these phenomena are intertwined with bias leading to interactions that result in IP and high levels of IP leading to burnout (Fig. 1). To truly understand each and work toward change, the interplay between these topics must be examined in the spectrum of causality. The interconnection of these phenomena contains drivers and repercussions creating a substantial negative cycle that demands simultaneous multimodal solutions to significantly influence on the culture within health care. To understand the impact each has on the other, and work toward meaningful change at a systemic level, it is critical to first understand each challenge individually.

### Bias

Unconscious, or implicit, bias is an intrinsic part of how we interact with society as a whole. These biases are based on previous experiences and current attitudes, both of which impact our perceptions. Subsequently, this affects our interpersonal interactions, behavior, and decision making.^2^ From an evolutionary standpoint, these unconscious biases developed initially to ensure the survival of the human race.

The ability to quickly ascertain whether a situation was dangerous or safe based on previous encounters was necessary.^3^ Today, these associations and mental short cuts persist and help us categorize scenarios and interactions and respond. Just as in society, both unconscious and implicit biases exist in health care, as do explicit and conscious biases. These biases impact the way in which we interact with colleagues, can influence the way in which we approach certain situations, and can have a direct effect on patient care.

### Impostor phenomenon

IP was described in 1978 by Drs. Clance and Imes as the internal feeling of “intellectual phoniness”; wherein individuals, particularly high achieving women, believed they had fooled others and were not as bright as others believed, despite outstanding accomplishments.^4^ The term IP subsequently described high achievers with traits and behaviors resulting in difficulty internalizing their successes. Those experiencing IP will express awareness of how others see them, but believe these accolades are not earned.^5,6^ Despite external opinions relating success, those with IP lack the confidence they can replicate past success, therefore, maintaining the perceived “ruse” of success becomes their goal.

This often leads to a significant amount of fear that can be associated with a focus on self-monitoring behaviors,^6–8^ and can lead to anxiety, depression, decreased job satisfaction, lack of confidence, and an inability to achieve goals. Since being described, IP has been identified in many high-achieving professions across a variety of fields, including health care and associated with significant anxiety, psychological distress, and these and other mental health consequences subsequently lead to burnout.^9^

### Burnout

Burnout is defined as a syndrome of exhaustion, cynicism, and decreased effectiveness at work. Since its initial description in 1974, burnout has been noted to impact workers in various fields; however, it is extremely prevalent in fields with intense interactions with people, such as health care. The first study of burnout among U.S. physicians across all specialties was performed in 2011. Of the 7288 participating physicians, 45% reported at least one symptom of burnout.^10^

Many potential contributors to burnout exist, including excessive workloads, inefficient work processes, clerical burdens, lack of input or autonomy for physicians relating to issues that directly impact their work lives, organizational support structures, and leadership culture. Individual physician-specific factors have also been found to play a role, and higher rates of burnout are reported in female and younger physicians.^11^

The escalation of impact of burnout and effect on mental health in health care during the COVID-19 pandemic must not go unrecognized in this discussion.^12^ The high burden of workload, combined with the distress related to many uncertainties throughout this pandemic, carries potential for profound increases in the effect of burnout and possible degradation of productivity, attrition of health care practitioners, and long-term impact on the medical workforce.^13^

### Interplay

The feelings of IP can be fueled by intrinsic and extrinsic causes. Initial research by Clance and Imes in 1978, and Harvey and Katz in 1985, found a significantly higher prevalence of IP for professional women.^4,14^ Further research discovered that IP can be prevalent in both men and women.^8,15,16^ IP is linked to family background, specifically in individuals who are the first in their family to exceed expectations for success. It has also been associated with careers where objective measures of success are not always aligned with the quality of the product or work.^6,14^ In addition, within professions where one gender predominates, individuals of the opposite gender are more likely to display impostor behavior.^14^

Extensive data demonstrate that women in medicine face many extrinsic challenges and barriers such as a lack of mentors,^17^ explicit discrimination, and gender bias, pay disparities,^18^ and a lack of recognition for equal work. A study in the *Journal of Clinical Oncology* reported women were less likely than men to be introduced by formal title when presenting at an international conference.^19^ Similarly, gender disparities in introductions are reported for grand rounds introductions.^20^ These examples of implicit, and sometimes explicit, biases can ultimately lead to feelings of IP. Synergistically with the existing barriers, these biases ultimately derail a woman physician's career.^21^

These implicit biases have a direct impact on the confidence of the individual and can result in planting the seeds of doubt that eventually fuel further feelings of IP. As those with IP lose self-confidence, internalize failures, and hyper-focus on mistakes, stress and anxiety become internalized and externalized. Owing to this, they try to decrease the impact of both by working harder, seeking perfection, and overstretching themselves.^6,15,22^ Individuals manifesting IP tendencies place an extreme emphasis on perfection and effort, and as a result, often self-inflict standards for unachievable unrealistic goals. When those goals are not achieved, or the individual is not acknowledged for their successes, these feelings are accentuated, resulting in an endless cycle of workaholic behaviors that then lead to exhaustion and burnout.^8,15,23^

In addition, this may lead to increasing pressure to perform in public situations, thus further increasing the stress, and causing those with IP to stop finding joy in their work, and turn down new opportunities.^5,8,15^ For those who do accept opportunities such as a new role or position, IP may be particularly prevalent. Kets de Vries astutely note that in reality, we are all imposters to some degree. “We play roles on the stage of life, presenting a public self that differs from the private self...Displaying a facade is part and parcel of the human condition.”^8^ However, for those who have IP, these feelings of being an impostor may not go away.

The prevalence of the IP in higher education is also well documented. Research has found IP tendencies in student, faculty, and staff. Hutchins noted that those attracted to higher education, and the current work environment in these settings, align closely to the factors that contribute to the development of impostor tendencies.^24^ IP is more common in those with traits of conscientiousness, achievement orientation, perfectionistic expectations, and those working in stressful and highly competitive professions.

A study examining the levels of burnout and IP in medical students found that female gender was significantly associated with IP with more than double the percentage of females displaying IP compared with their male counterparts. The study also reported IP was significantly associated with burnout components of exhaustion (*p* = 0.045), cynicism (*p* = 0.004), emotional exhaustion (*p* = 0.018), and depersonalization (*p* = 0.015). The study concluded that almost a quarter of male medical students and nearly half of female students experienced IP, which was significantly associated with burnout indices.^25^

Another study examined the incidence of IP and burnout in general surgeons and surgery residents. The majority of the surgeons and residents surveyed were male, Caucasian, and married. This study found residents scored significantly higher than faculty in almost half of the Clance Impostor Phenomenon Scale (CIPS) and the overall CIPS score was also significantly higher in trainees. Reported burnout was comparable between trainees and faculty.^26^

Regarding bias, the pervasive existence of explicit and implicit racial bias is described in the literature, as well as its impact on patient care. Of 347 medical student interviewees, 21% confirmed they experienced discrimination on the interview trail, with gender, age, race, religion, and sexual orientation all sources of discrimination. Those reporting bias were more likely to be Latinx, and only 4% of those who experienced discrimination reported the incident to the institution where it occurred.^27^ In a 2020 NEJM Catalyst survey with 553 respondents, ∼50% of clinicians and staff reported being impacted by interpersonal racism, and >50% report patients are impacted by disparities in care delivery.^28^ The high rates of reported discrimination and/or racism raise further concern for potential impact on IP.

Some minority groups may be more susceptible to IP. A study in 2013 from the University of Texas at Austin found that, per census definitions of race, Asian Americans were more likely than African Americans or Latino Americans to experience impostor feelings. African Americans reported higher minority status stress. Both minority status stress and impostor feelings were assessed as possible predictors of mental health. The study found that impostor feelings predicted mental health problems more strongly than stress related to one's minority status.^29^ Furthermore, black physicians have increased risk of burnout, which has been partially attributed to discrimination,^30^ both of which are associated with IP.

Numerous studies note the direct impact on patient care due to implicit bias.^2,31^ Adding to literature, the COVID-19 pandemic has both highlighted and worsened racial health disparities.^28,30^ On literature review, six studies found that higher implicit bias was associated with disparities in treatment recommendations, expectations of therapeutic bonds, pain management, and empathy.^31^ In addition, under the burden of burnout, biases may manifest at higher levels, causing increasing disparities in patient care.^30^

Physician burnout has been linked to higher mortality ratios in hospitalized patients, self-reported errors, and turnover.^32^ Studies have also suggested a link between burnout and a decrease in the amount of time physicians spend providing clinical care to patients.^10,33^ Thus the implicit bias that can contribute to feelings of IP and lead to burnout may also directly impact the care provided to patients and health care outcomes; given reported increasing concern for burnout due to COVID-19, the stakes have never been higher.^30^

There is a need for future research and implementation of multimodal solutions at a systemic level to remedy this problem. It is essential that research focused in any of these areas also assesses the possible effect of impacting the other phenomena, either by worsening or improving on them. Implicit bias training and longitudinal follow-up have been shown to be an effective way to mitigate stereotypes and unconscious biases that exist at an individual and at a system level.^34^ Many institutions are more open in discussing wellness and self-care; however, it is essential to intentionally and methodically implement real changes at the system level that directly address the underlying etiologies that lead to burnout.

## Conclusion

It is inevitable that when implicit bias and burnout are pervasive in our health care system, those impacted will be more likely to develop feelings of IP, and this may then lead to burnout, if not already present. The interaction of bias, burnout, and IP carries severe costs for the health care workforce and patient care. We can no longer blindly try to fix one component of these, but we must bravely step forward with cultural overhauls addressing all three together. Our future profession and patients require it.

## Abbreviations Used

IP: impostor phenomenon
CIPS: Clance Impostor Phenomenon Scale
